# IL10 (-1082 G>A, rs1800896) gene polymorphisms are associated with oxidative stress in sickle cell disease patients in Uganda

**DOI:** 10.1186/s40659-025-00639-w

**Published:** 2025-09-03

**Authors:** Cissy B. Namuleme, Charles D. Kato, Dennis M. Kasozi

**Affiliations:** 1 Department of Biotechnical and Laboratory Sciences, School of Biosecurity, College of Veterinary Medicine, Animal Resources and Biosecurity, Makerere University, P.O. Box 7062, Kampala, Uganda; 2 Department of Biochemistry and Systems Biology, School of Biosciences, College of Natural Sciences, Makerere University, P.O. Box 7062, Kampala, Uganda

**Keywords:** Sickle cell disease, Oxidative stress, Interleukin 10 gene polymorphisms, TNFαβ gene polymorphisms

## Abstract

**Background:**

Sickle cell disease (SCD) is characterised by chronic oxidative stress. However, there is limited information on how polymorphisms in cytokine genes influence oxidative stress in SCD patients. The study aimed to determine the effect of Interleukin gene (*IL-10*) and Tumor Necrosis Factor (*TNF*_*αβ*_) polymorphisms on oxidative stress and cytokine levels in SCD patients from Mulago hospital.

**Methods:**

A case control study with cross-sectional sample size of 163 SCD patients and 189 healthy controls was carried out. The extent of oxidative stress was quantified using Malondialdehyde (MDA) by spectrophotometry. Levels of IL-10 and TNF-α were measured using the Enzyme-Linked Immuno-Sorbent Assay (ELISA). The Amplification Refractory Mutation System polymerase chain reaction (ARMS-PCR) assay was used to genotype IL10-1082 A > G, (rs1800896), IL10-819 C > T (rs1800871) and TNF-α-308G > A (rs1800629) and TNF-β + 252 A > G (rs909253) gene polymorphisms.

**Results:**

Samples showed significantly (*P* = 0.0063) higher median plasma levels of MDA in SCD patients (2.756µM) than healthy controls (2.364µM). A similar trend was observed with significantly (*P* < 0.0001) higher median plasma levels of IL-10 in SCD patients (20.37pg/ml) than healthy controls (7.5pg/ml). The most frequent genotype for IL-10 (-1082, rs1800896) gene polymorphism was heterozygous GA (62.6%). No significant association between IL10 (-1082G > A, rs1800896) gene polymorphisms and SCD was observed (OR = 1.08, 95% CI = 0.54–2.14, *P* = 0.87). Yet, IL10 homozygous GG (-1082, rs1800896) (22.12pg/ml) that was found to be significantly associated (*P* = 0.0234) with increased plasma levels of IL-10 as compared to heterozygous genotype (GA) (13.94pg/ml) in SCD patients. Similarly, higher levels of MDA were found to be significantly (*P* < 0.0001) associated with homozygous GG at IL-10 (-1082, rs1800896). The most frequent and only reported genotype for TNF-α/β gene polymorphisms were heterozygous GA, thus no associations were described.

**Conclusion:**

In conclusion, our results suggest that the IL10 (-1082 G > A, rs1800896) gene polymorphism is associated with increased oxidative stress and IL-10 cytokine level in Ugandan SCD patients.

## Introduction

Sickle cell disease (SCD) is a neglected major cause of infant mortality in Africa [1, 2]. Sub-Saharan Africa has a reported 75% of the global SCD incidence with a predicted increase by 2050 [3]. Uganda has a 13·2% prevalence for sickle cell trait and 0·8% for SCD in children [4]. SCD is characterised by intravascular haemolysis and vaso-occlusion events, the key promoters of chronic inflammation and redox instability [5, 6]. Consequently, causing gradual small and large vessel vasculopathy, reperfusion injury and end-organ damage [7]. Malondialdehyde (MDA), a known stable biomarker for oxidative stress has been reported to be high in steady-state and increased in severe SCD [8]. However, low plasma levels of IL-10, an anti-inflammatory cytokine have been reported during crisis and high in steady state SCD patients [9]. Decreased levels of IL-10 have also been associated with osteomyelitis in patients [9] suggesting a protective role of IL-10. An increased production in IL-10 levels has been associated with GCC alleles unlike the mutants ATA for − 1082/-819/-592 IL-10 haplotypes in severe malaria patients [10]. The heterogeneity in the inflammatory profile of SCD may also be due to differences in populations in addition to genetics [11]. The study aimed to determine the effect of *IL-10 (-1082G > A*,* rs1800896)* and *(-819 C > T*,* rs1800871)*, and *TNF-α-308G > A (rs1800629)*,* TNF-β + 252 A > G (rs909253)* polymorphisms on oxidative stress and IL10 levels in Ugandan SCD patients from Mulago hospital.

## Materials and methods

### Study population

This study was approved by Makerere University School of Health Sciences Research and Ethics Committee (MAKSHSREC- 2021-90) and a waiver of informed consent to use study participants’ samples was obtained. A sample size of 150 in each group with a 90% power to detect a difference between MDA means of 0.57 [8] with a significance level (alpha) of 0.05 (two-tailed) was determined using GraphPad StatMate 2.00 statistical software. A total of 352 participants were sampled in this study and among them were, 163 SCD patients from Mulago hospital sickle cell clinic and 189 healthy controls from Nakasero Blood bank, Central Uganda. Using the sickle cell anaemia genotyping test [12] SCD patients as cases and healthy controls were identified. Healthy controls that are heterozygous with a sickle cell trait were excluded from the study.

### Determination of malondialdehyde, IL-10 and TNFα concentrations

Whole blood (~ 5mL) was centrifuged at 1000×g for 10 min to obtain 1000 µl of plasma and 500 µl of buffy coat. The plasma obtained was analysed for MDA levels to determine the extent of oxidative stress in SCD patients and controls using methods as described [13]. Plasma levels of IL-10 and TNF_α_ were quantified in both SCD patients and healthy controls using Enzyme Linked Immunosorbent Assay (ELISA) kit (BD Biosciences, Pharmingen, San Diego, USA), following the manufacturer’s instructions.

### Genotyping for sickle cell anaemia, IL-10 and TNF αβ gene polymorphisms

DNA was obtained from the buffy coat layer using methods described by Iranpur-Mobarakeh et al. [14]. Sickle Cell Anemia genotyping was carried out as described [12]. Cytokine gene polymorphisms were determined by Amplification Refractory Mutation System-PCR (ARMS-PCR) as described [15]. The ARMS-PCR technique was for genotyping cytokine gene polymorphisms: IL10-1082 A > G, (rs1800896), IL10-819 C > T (rs1800871) and TNF-α-308G > A (rs1800629), TNF-β + 252 A > G (rs909253) gene polymorphisms. The primer sequences are in Table 1. A2% agarose gel electrophoresis containing 0.5 mg/ml of ethidium bromide was used to view the amplified products, against a 50-bp ladder *(*Thermo Fisher Scientific, Waltham, MA, USA).

**Table 1:** Primer sequences

Primer name	Primer sequence	Product size
**Wild type β-globin gene**	AS : 5’-ATG GTG CAC CTG ACT CCT GA-3	517 bp
	CP517: 5’CCC CTT CCT ATG ACA TGA ACT-3	
**Mutant** **β-globin gene**	AS: 5’-CAG TAA CGG CAG ACT TCT CCA-3’	267 bp
	CP267 (5’-GGG TTT GAA GTC CAA CTC CTA-3’	
**IL-10** **(-1082)**	Generic primer (antisense): 5’-CAGTGCCAACTGAGAATTTGG-3’	258 bp
	Primer G (sense): 5’CTACTAAGGCTTCTTTGGGAG-3’	
	Primer A (sense): 5’ACTACTAAGGCTTCTTTGGGAA-3’	
**IL-10** **(-819)**	Generic primer (antisense): 5’-AGGATGTGTTCCAGGCTCCT-3’	233 bp
	Primer C (sense): 5’-CCCTTGTACAGGTGATGTAAC-3’	
	Primer T (sense): 5’-ACCCTTGTACAGGTGATGTAAT-3’	
**TNF-α-308**	Generic primer (antisense): 5’-TCTCGGTTTCTTCTCCATCG-3’	154 bp
	Primer G (sense):5’-ATAGGTTTTGAGGGGCATGG-3’ (TNF1 allele)	
	Primer A (sense): AATAGGTTTTGAGGGGCATGA-3’ (TNF2 allele	
**TNF-β** **(Intron1 + 252)**	Generic primer (antisense): 5’-AGATCGACAGAGAAGGGGACA-3’	94 bp
	Primer G (sense): 5’-CATTCTCTGTTTCTGCCATGG-3’	
	Primer A (sense): 5’-CATTCTCTGTTTCTGCCATGA-3’	
**Internal control HGH**	Forward primer: 5’-GCCTTCCCAACCATTCCCTTA-3’	429 bp
	Reverse primer: 5’-TCACGGATTTCTGTTGTGTTTC-3’	

**IL10: Interleukin 10, TNFαβ: tumor necrosis factor, HGH: human growth hormone**

### Statistical analysis

Deviation from normality was tested using the D’Agostino-Pearson omnibus normality test. Age was expressed as mean ± SD for each study group. Using the Mann-Whitney test, comparison of MDA levels between groups and sex for SCD was achieved. The Kruskal-Wallis test was used to compare median MDA levels across different age groups in SCD. The study groups were tested for Hardy-Weinberg equilibrium by comparing the expected with the observed genotype frequencies. The Chi-square (χ2) test was used to compare gene frequencies between SCD and the Healthy group. Association between genotypes with MDA and IL-10 levels was achieved using the Mann-Whitney test. All analyses were calculated with Graph Pad Prism 5.0 Software (Graph Pad Software, La Jolla, CA, USA). The level of statistical significance was set as *P* < 0.05.

## Results

### Baseline characteristics of the study population

**Fig. 1:**
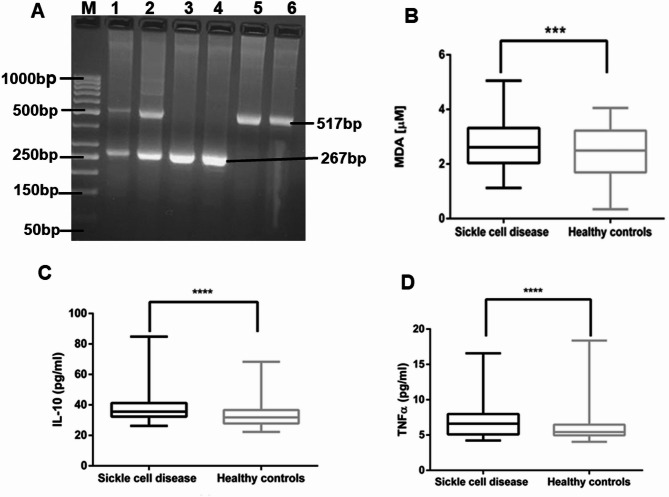
Levels of MDA, IL10 and TNFα between SCD patients and healthy control groups. **(A)** A representative gel showing results of sickle cell anaemia genotyping. Lane 1; DNA ladder, lanes 2 & 3; HbAS bands, lanes 4 & 5; HbSS bands, lanes 6 & 7; HbAA bands, **(B)** MDA **(C)** IL10 and TNF **(D)** levels differed between sickle cell disease and healthy controls. MDA: Malondialdehyde, IL10: Interleukin − 10, SCD: Sickle cell disease. HbSS: sickle cell anemia, HbAA: normal Hemoglobin, HbAS: heterozygous sickle cell individuals (carriers). “*” indicates a difference between SCD patients and healthy control groups. ^*^P < 0.05, ^**^P < 0.01,^***^P < 0.001, **** *P* < 0.0001. Data analysis by Mann-Whitney test. Outliers not shown

Of the 163 SCD patients, 66 (40.5%) were females. Of the 189 healthy individuals, 45 (23.8%) were females. The average age for the SCD group was 9.8 ± 0.64 years, while that of the control group was 27 ± 0.67 years. SCD was more prevalent in patients under 5 years (45, 27.6%) and children aged 5–14 years (82, 50.3%) as compared to older patients aged above 15 years (36, 22.1%). This study used a cross-sectional sample size, so the patient and control groups were not age/sex matched. After genotyping (Fig. 1A), a total of 9 (4.7%) individuals were identified as carriers for the sickle cell gene and excluded from the healthy control group.

### Levels of oxidative stress and IL-10 differed significantly between SCD patients and healthy controls

Oxidative stress was determined through the plasma analysis of MDA levels in SCD patients (*N* = 163) and controls (*N* = 189). The D’Agostino-Pearson test results for MDA (K2 = 9.483, *p* = 0.0087) and IL-10 (K2 = 78.82, *p* < 0.0001) indicated that the data for both analytes are not normally distributed. Results showed that median plasma levels of MDA were significantly higher (*P* = 0.0002, Mann-Whitney test, Fig. 1B) in SCD patients (2.756µM) as compared to healthy controls (2.364µM). The role of cytokines in the modulation of SCD was demonstrated through the assay of IL-10 cytokines in the plasma of both SCD patients (*N* = 163) and healthy controls (*N* = 189). The detection limits for the assays were 14.2pg/ml (IL-10). Median plasma levels for IL-10 were significantly higher (*P* < 0.0001, Fig. 1C, Mann-Whitney test) in the plasma of SCD patients (20.37pg/ml) as compared to healthy controls (7.5pg/ml). On the other hand, plasma levels of TNF-α were not significantly different between SCD patients and healthy controls (Fig. 1D).

### Prevalence of IL-10, TNF-α and TNF-β genes polymorphisms in SCD patients and healthy controls

**Fig. 2:**
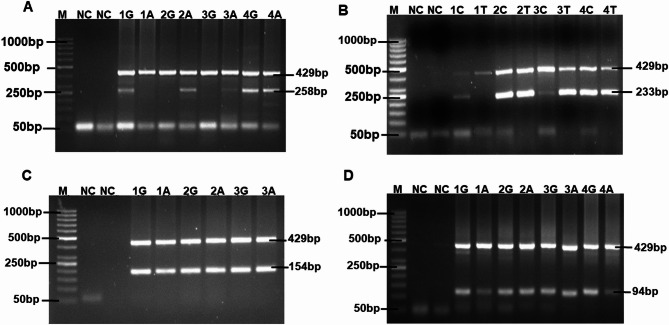
ARMS-PCR of *IL-10* and *TNFα/β* genotypes. Agarose gel electrophoresis of the ARMS PCR assay products. The 429 bp amplification product of the HGH control was present in all lanes, showing optimal amplification. M = 50 bp ladder, NC = Negative control. **(A**) IL-10 (-1082 > G/A): Lane 1G- 1 A = Homozygous GG; Lanes 2G- 2 A and 3G- 3 A = Homozygous AA and Lane 4G- 4 A = Heterozygous GA for IL-10 (-1082 G > A) polymorphism. **(B**) IL-10 (-819 C > T): Lane 1 C- 1T = Homozygous CC; and Lanes 2 C- 2T, 3 C- 3T and 4 C- 4T = Heterozygous CT for IL-10 (-819 C > T) polymorphism. **(C**) TNF-α (308 > G/A) genotypes from ARMS-PCR amplification. From left to right: NC = Negative control; Lanes 1G- 2 A; 3G- 4 A and 5G- 6 A = Heterozygous GA for TNF-α (308 > G/A) polymorphism in sickle cell disease patients. **(D**) TNF-β (Intron1 + 252 > G/A) genotypes from ARMS-PCR amplification. From left to right: NC = Negative control; Lanes 1G- 2 A; 3G- 4 A and 5G- 6 A = Heterozygous GA for TNF-β (Intron1 + 252 > G/A) polymorphism in sickle cell disease patients

The genotypes for IL-10 (-1082 > G/A, rs1800896) polymorphism were deduced from the presence or absence of a 258 bp amplicon specific for either G or A and both alleles (Fig. 2A). A 429 bp amplicon of the HGH as internal control was also observed in the gel, indicating optimal amplification of sample DNA of the study participants.

Our study shows that heterozygous GA was the most common genotype for IL-10 (-1082 G > A, rs1800896) polymorphism in both patients (62.61%) and healthy controls (64.3%). Similarly, 35.65% of SCD patients had a homozygous GG genotype while the control group scored 33.9%. The least genotype observed for IL-10 (-1082 G > A, rs1800896) was homozygous AA, with 1.74% in patients and 1.8% in healthy controls (Table 2). However, statistical analysis did not yield any significant (*P* < 0.05, Chi-square test) difference when IL-10 (-1082 G > A, rs1800896) genotype frequency was compared in patients and healthy controls. There was also no significant association between IL-10 (-1082 G > A, rs1800896) genotypes, GG (OR = 1.08, 95% CI = 0.54–2.14, *P* = 0.87); GA (OR = 0.93, 95%CI = 0.48–1.82, *P* = 0.87); AA (OR = 0.97, 95%CI = 0.11–14.34, *P* > 0.9999, Fisher’s exact test) and SCD. The G allele for IL-10 (-1082 G > A) polymorphism was more frequent in the study population as compared to the A allele.

For IL-10 (-819 C > T, rs1800871) polymorphism, genotypes were deduced from the presence or absence of a 233 bp amplicon specific for either C or T and both alleles (Fig. 2B). The most frequent genotype for IL-10 (-819 C > T, rs1800871) polymorphism in both SCD patients (99.13%) and healthy controls (100%) was heterozygous CT. The TT genotype was absent in the study population, while the CC genotype was present in the patient’s group (0.87%) but not in the healthy control group (0%) (Table 2). The genotypes for IL-10 (-819 C > T, rs1800871) polymorphism did not yield any significant (*P* > 0.9999, Fisher’s exact test) association with SCD. The allele frequency for IL-10 (-819 C > T) polymorphism was comparable between C and T alleles in the study population.

**Table 2:** Genotypic and allelic frequency distribution of IL-10 polymorphisms

Cytokine gene Polymorphism	Sickle cell patients			Healthy controls		Statistical analysis *P*-value (OR, 95% CI)
	Genotypic frequency *N* (%)	Allelic frequency		Genotypic frequency *N* (%)	Allelic frequency	
IL-10 (-1082G > A)						
GG	41 (35.65)	G = 0.6696		19 (33.9)	G = 0.6607	0.87 (1.08, 0.54–2.14)
GA	72 (62.61)	A = 0.3304		36 (64.3)	A = 0.3393	0.87 (0.93, 0.48–1.82)
AA	02 (1.74)			01 (1.8)		> 0.99 (0.97, 0.11–14.34)
IL-10 (-819 C > T)						
CC	01 (0.87)	C = 0.504		00 (0)	C = 0.5	> 0.99
CT	114 (99.13)	T = 0.496		56 (100)	T = 0.5	> 0.99 (0.00, 0.00- 18.48)
TT	00 (0)			00 (0)		

OR = odds ratio; CI = confidence interval

The genotypes for TNF-α (308 > G/A, rs1800629) and TNF-β (Intron1 + 252 > G/A, rs909253) polymorphisms were deduced from the presence or absence of a 154–94 bp amplicon specific for either G or A, and both alleles, Fig. 2C and D, respectively. Results from our study showed heterozygous GA (100%) as the most frequent and only genotype present in our study population for both TNF-α (308 > G/A, rs1800629) and TNF-β (Intron1 + 252 > G/A, rs909253) polymorphisms.

### Association between IL10 genotypes with plasma levels of malondialdehyde and IL-10

**Fig. 3:**
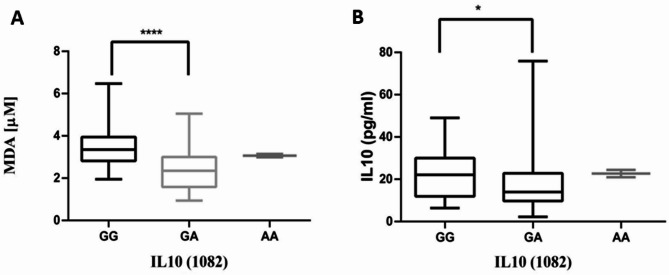
MDA and IL10 levels Between IL-10 genotypes. (**A**) MDA and IL10 (**B**) levels differed between GG, GA and AA in patients with SCD. MDA: Malondialdehyde, IL10: Interleukin − 10, SCD: Sickle cell disease. “*” indicates a difference between SCD patients and healthy control groups. ^*^P < 0.05, ^**^P < 0.01,^***^P < 0.001, **** *P* < 0.0001. Data analysis by Mann-Whitney test. Outliers not shown

Comparisons between IL-10 (-1082 GG vs. GA, rs1800896) genotypes with plasma levels of MDA yielded a significant difference (Mann-Whitney test, *P* < 0.0001) in SCD patients (Fig. 3A). Higher median levels of MDA were observed in patients with GG (3.35µM) genotype as compared to those with GA (2.30µM) genotypes. To determine whether IL-10 (-1082 G > A, rs1800896) gene polymorphism was associated with changes in IL-10 levels, plasma concentrations of IL-10 were compared across IL-10 (GG vs. GA) genotypes in SCD patients. There was a significant difference (Mann-Whitney test, *P* = 0.0234, Fig. 3B) in IL-10 levels across the genotypic groups. Homozygous GG (22.12pg/ml) genotype showed higher median plasma levels of IL-10 as compared to GA (13.94pg/ml) genotypes. Comparisons were not made for other cytokine gene polymorphisms, including IL-10 (819 > C/T, rs1800871), TNF-α (308 > G/A, rs1800629), and TNF-β (Intron1 + 252 > G/A, rs909253), because their genotypes lacked the required heterogeneity in the sampled population.

## Discussion

Oxidative stress and inflammation contribute significantly to the pathophysiology of SCD [16]. Our study found that the *IL10 (-1082G > A*,* rs1800896)* gene polymorphism is associated with increased levels of IL10 and oxidative stress in SCD patients in Uganda. The key sources of oxidant injury in SCD include increased levels of cell-free hemoglobin following chronic hemolysis, mechanisms leading to vaso-occlusion ischemia-reperfusion and inflammation [7]. Similarly, activation of inflammatory cells and their signalling pathways culminates into the release of molecules that drive the inflammatory state in SCD such as cytokines, chemokines and growth factors [7]. TNFα is a pro-inflammatory cytokine and is generated as an early consequence of ischemia reperfusion [17] with increased levels being detected during pain crisis. While IL-10 cytokine is known to limit the production of pro-inflammatory cytokines such as TNF-α, it is also found to be elevated in steady-state SCD patients [18]. Recently, polymorphisms in cytokine genes have also been associated with increased cytokine production and consequently, a heterogeneous clinical outcome of SCD in patients with stroke, leg ulcers, splenic sequestration and end-organ damage among others [19]. Our current research aimed at studying the relationship between SCD pathogenesis and the key modifiers of disease severity like oxidative stress, cytokine levels and cytokine gene polymorphisms in the Ugandan population.

The median plasma levels of MDA were significantly higher in SCD patients as compared to healthy controls. These results were consistent with previous studies, reporting increased levels of MDA in severe SCD (with pain crises) and steady state patients (without pain crises) as compared to healthy controls [20, 21]. However, contradicting results for MDA levels in SCD when compared to healthy controls have also been reported [22]. The extent of oxidative damage measured as MDA in patients was higher due doubled generation of reactive oxygen species during intracellular catabolism and from the pathological events of a sickled red blood cell such as increased hemoglobin autoxidation [23] increased release of cell free hemoglobin [24] and pro-inflammatory molecules [5]. Likewise, our comparison of median MDA levels across sex and different age groups in SCD patients did not yield any significant difference, attributing to SCD pathogenesis was the primary source of increased oxidative damage [25]. Oxidative damage in patients usually results into endothelial dysfunction, vaso-occlusive pain, early organ damage and continuous inflammation [16]. This study also found significantly higher plasma levels of IL-10 in SCD patients as compared to healthy controls. This finding agreed with previous results that showed increased serum levels of IL-10 in steady-state SCD and its possible inhibition of both humoral and cell-mediated immune functions [18, 23]. The SCD patients included in this current study were asymptomatic cases. Contrary to our results, no significant difference in serum levels of IL-10 between SCD patients and healthy controls was reported in Brazil [26]. Nonetheless, being an anti-inflammatory molecule, IL-10 inhibits the synthesis of TH1cytokines, such as TNF-α, IL-1, IL-6, and IL-8. The plasma levels of TNF-α were not significantly different between SCD patients and healthy controls [27].

The production of IL-10 can be controlled at the transcriptional level consequently, the 3 bi-allelic single-nucleotide polymorphisms from the transcription start site, at positions − 1082 G > A, -819 C > T and − 592 C > A have been studied [28]. The genotype frequency differences reported for IL-10 (-1082G > A, rs1800896) in this study were not statistically significant despite similar group sizes between SCD patients and healthy controls. This finding was in agreement with previous studies [26]. However, our sample population showed heterozygous GA as the most frequent genotype for IL-10 (-1082G > A, rs1800896) polymorphism followed by GG. Our results were similar to those for IL-10 (-1082G > A, rs1800896) genotype frequency reported from India [18] and Brazil [19] in SCD patients. However, a different trend of AA as the most frequent genotype, followed by GA and GG has been reported for (-1082G > A, rs1800896) polymorphism in Kenya [10] and in Brazil [29] respectively which could be due to differences in the population structure. The genotype and allele frequencies of the *IL10 (-1082G > A*,* rs1800896)* and *IL10 (-819 C > T*,* rs1800871)* corroborate with those reported from seven African sub-populations including the African Caribbean in Barbados (ACB), African ancestry in Southwest US (ASW), Esan in Nigeria (ESN), Gambian in Western Division (GWD), Gambia, Luhya in Webuye, Kenya (LWK), Mende in Sierra Leone (MSL) and the Yoruba in Ibadan, Nigeria (YRI) in the 1000 genomes project. The source of variation in findings may be attributed to population heterogeneity and sampling methods used. Similarly, the IL-10 (-819 C > T, rs1800871) genotypic frequency showed no significant difference when compared between SCD patients and healthy controls. This finding was agreement with previous studies [10, 19, 26]. We did not investigate IL-10 -592 position because it was reported to be in LD with the − 819 position. The alleles for − 819 C > T and − 592 A > C are inherited together [28] thus, in our current study, the *IL-10 (-819 C > T*,* rs1800871)* SNPs was characterized. The most frequent genotype in our SCD study population was heterozygous CT. The mutant genotype TT was absent in the sampled SCD population. A similar trend for IL-10 (-819 C > T, rs1800871) genotype frequency was reported [10, 19] but a variation in results is due to population structure and sampling methods used.

Plasma changes in IL-10 levels were significantly different across the IL-10 (-1082G > A, rs1800896) gene polymorphism with homozygous GG genotype showing higher median levels of IL-10 as compared to GA + AA genotypes in SCD patients. Consistent with this current study, individuals positive for the mutant allele A at position − 1082 for IL-10 had significantly higher IL-10 production as compared to those negative for the A allele [30, 31]. This association was independent of the (-819 C > T, rs1800871) polymorphisms. Alternatively, other studies [10] also reported that the GCC haplotype was associated with increased IL-10 production unlike the ATA haplotype in patients with severe malaria [32]. Enhanced binding of transcriptional factors that promote higher IL-10 production maybe favoured by the presence of GCC haplotype unlike the ATA haplotype that may prime enhanced binding sites for repressors and thus reduced IL-10 production [10]. As anti-inflammatory molecule, IL-10 inhibits the production of type-1 cytokine that drive the inflammatory state in SCD, and changes in the cytokine can be used in vaso-occlusion crisis prognosis [23].

Similarly, plasma levels of MDA were found to be significantly different across IL-10 genotypes with homozygous GG genotype showing higher levels of MDA as compared to GA + AA genotypes for *IL-10 (-1082 G > A*,* rs1800896)* gene polymorphism in SCD patients. However, molecular mechanisms linking IL-10 genotype to oxidative stress remain unclear. A wide range of transcription factors and receptors, like the nuclear factor-κB (NF-κB) and activator protein-1 (AP-1) [33], which regulate the expression of many genes, including those involved in the production of both pro-inflammatory and anti-inflammatory cytokines [34]. However, little research has been done on the possible inter-relationship between oxidative stress and inflammation in SCD.

This study had some limitations. First, samples were not representative of the entire population since they were obtained from the Mulago hospital located in the central region whereas Uganda is an ethnically diverse country. Secondly, samples were not age/sex matched and multivariate analyses could not be performed. Thirdly, besides IL-10 (-1082G > A, rs1800896), IL-10 (-819 C > T, rs1800871), several other SNPs in other cytokines may affect the relation between cytokine levels and oxidative stress in SCD patients. However, this is the *first study* in Uganda that provides useful insights into the association between cytokine gene polymorphisms, cytokine levels and oxidative stress in SCD patients. In further research, a multi-site study with diverse ethnic participants may be necessary to investigate the association between SNPs, cytokine levels and oxidative stress in Ugandan SCD patients.

## Conclusion

Collectively, results from this study suggest that SCD is associated with increased IL-10 cytokine and plasma levels of oxidative stress. Also, IL-10 (-1082 > G/A, rs1800896) gene polymorphism was associated with changes in IL-10 levels but the genotype frequency differences were not statistically significant despite similar group sizes between SCD patients and healthy controls.

## Data Availability

All data generated or analysed during this study are included in this published article. The UniProt accession numbers for IL-10, TNF-α and TNF-β were P22301, P01375 and P01374 respectively.

## Abbreviations

ARMS: PCR Amplification Refractory Mutation System PCR
ELISA: Enzyme Linked Immunosorbent Assay
EDCTP2: European and Developing Countries Clinical Trials Partnership
HbAA: Homozygous normal hemoglobin
HbAS: Heterozygous sickle hemoglobin
HbSS: Homozygous sickle cell recessive hemoglobin
HGH: Human growth hormone
IL-10: Interleukin- 10
MDA: Malondialdehyde
MAKSHSREC: Makerere University School of Health Sciences Research and Ethics Committee
MAPRONANO: Materials, Product Development and Nanotechnology
ROS: Reactive Oxygen Species
SIDA: Swedish International Development Cooperation Agency
SCD: Sickle Cell disease
SCA: Sickle Cell Anemia
SNPs: Single nucleotide polymorphisms
TNF-α: Tumor Necrosis Factor Alpha
TNF-β: Tumor Necrosis Factor beta
